# Merits of bioactivity and products of *Dendrobium officinale* and other related species

**DOI:** 10.3389/fpls.2026.1864437

**Published:** 2026-06-25

**Authors:** Shaojun Wu, Jinchuan He, Yanli Zhang, Haihua Zhang, Xiaodan Zhang, Zongsuo Liang

**Affiliations:** School of Life Sciences and Medicine, Zhejiang Sci-Tech University, Hangzhou, China

**Keywords:** bioactivity, dendrobium genus, *Dendrobium officinale*, development trends, extraction method, product development

## Abstract

The genus Dendrobium is a renowned traditional Chinese medicinal plant and one of the most extensively studied and widely applied species within the Dendrobium genus, possessing significant medicinal value and industrial development potential. This paper reviews the resource distribution and quality correlation, development trends, chemical constituents, biological activities, clinical applications, and current product development status of *Dendrobium officinale* and other medicinal plants within the Dendrobium genus. It highlights the structural characteristics and pharmacological effects of key active components such as polysaccharides, alkaloids, and dibenzyl compounds, summarizes their mechanisms of action in immune regulation, antioxidant properties, and eye health protection, and supplements the influence of different origins and cultivation models on active components. The paper also provides an outlook on the current status of product development and future trends.

## 1 Introduction

The genus Dendrobium encompasses a wide array of species valued for their ornamental and medicinal properties, holding a preeminent position in Traditional Chinese Medicine (TCM), historically revered as the “Emperor of Herbs” and recorded as a superior-grade medicine in Shennong Bencao Jing for its efficacy in nourishing yin, clearing heat, benefiting the stomach, generating fluids, and moisturizing dryness and promoting fluid production.

In parallel, several other Dendrobium species are also utilized as the herbal medicine “Shihu”. Importantly, *Dendrobium officinale* accumulates a series of characteristic and exclusive metabolic constituents that are rarely found in most conventional medicinal plants, typically including unique dendrobium polysaccharides and specific dendrobium alkaloids, as well as bibenzyl derivatives. Meanwhile, only trace amounts of potential anti-nutritional components exist in *Dendrobium officinale*, and these weak anti-nutritional substances can be easily removed by conventional extraction and processing technologies. Such chemical characteristics endow *Dendrobium officinale* with high safety and excellent exploitable value, providing a solid theoretical basis for its further development of functional foods, health supplements and related products.

The cultivation of *Dendrobium officinale* in China is primarily concentrated in regions south of the Yangtze River, notably forming industrial clusters in Yunnan and Zhejiang. Geographical origin and cultivation conditions serve as decisive extrinsic factors that directly regulate secondary metabolite biosynthesis of *Dendrobium officinale*. Variations in altitude, light intensity, temperature and soil microenvironment across different habitats alter the synthesis and accumulation level of polysaccharides, alkaloids, bibenzyls, and flavonoids. Meanwhile, diverse artificial cultivation modes further reshape component profiles, resulting in distinct pharmacological performances and inconsistent material quality. Combined with genetic characteristics and post-harvest processing techniques, these factors collaboratively determine the final yield and active ingredient quality. Exploring the correlation between environmental factors and key metabolites in the biosynthesis process has long served as a core research focus for improving product quality and achieving sustainable resource exploitation.

Relevant literature was analyzed by bibliometric methods, with VOSviewer and CiteSpace adopted as the core analytical tools. The results reveal that the research hotspots in this field show a distinct evolutionary trend. Research priorities have gradually evolved from early species identification and authenticity verification to the isolation, purification and bioactivity screening of core components (predominantly polysaccharides). In recent years, relevant studies have further shifted to the exploration of action mechanisms underlying intestinal flora, network pharmacology, and specific signaling pathways such as nuclear factor-kappa B (NF-κB). These research findings provide scientific evidence for its traditional application in alleviating discomforts including dry mouth, thirst and blurred vision, and also facilitate its development in the fields of dietary supplements and functional products.

Despite the number of relevant research papers has increased significantly in recent years, most previous reviews only focused on individual components, single biological activity or regional cultivation experience, while neglecting an integrated overview across phytochemistry, multi-target pharmacological mechanisms and industrial product development. Moreover, existing summaries rarely systematically clarify the intrinsic correlation between habitat/cultivation patterns, chemical marker variation and differential bioactivities of Dendrobium species. In addition, ranging from mechanism exploration and biological activity verification to the research and development of standardized and circular products, this transformation process remains the major bottleneck restricting the development of the current field.

This review, therefore, aims to provide a comprehensive and updated synthesis of the current knowledge on medicinal Dendrobium plants. We will systematically summarize on their resource distribution, industrial layout, elaborate the structural characteristics, optimized extraction strategies, and multi-dimensional bioactivities of major active constituents, as well as their traditional efficacy and modern clinical progress. Furthermore, we comprehensively evaluate the current status, existing limitations and future developmental directions of Dendrobium-derived functional foods, health supplements and cosmetic products. This work not only clarifies the research frontier and unresolved challenges in this field, but also provides original insights for linking chemical structural characteristics, pharmacological mechanisms and industrial application, aiming to bridge the gap between fundamental phytochemical research, pharmacological verification and industrial transformation of this precious medicinal resource.

## 2 Statistical analysis of the bibliography

### 2.1 Analytical software and methods

Bibliometric statistics is a useful methodology derived from the junction of statistics, bibliometrics, and information science. It uses quantitative indicators, data mining, and statistical analysis to organize and analyze research subjects, create reviews, conduct academic evaluations, and provide objective data support (Wei et al., 2025).

VOSviewer and CiteSpace are two widely used bibliometric visualization analysis software, which can conduct in-depth analysis of data such as keywords, authors, research institutions and citations. Both tools are applicable to academic research in specialized fields such as biology and pharmacology, but they have distinct characteristics in terms of functional positioning and technical features. As a free and open-source software, VOSviewer’s core advantage lies in its ability to convert raw data into intuitive network visualization maps. CiteSpace is a classic software program that excels at creating cluster maps, timeline maps, and word-frequency maps. It effectively identifies evolving research hotspots, emerging frontiers, core literature frameworks and interdisciplinary cooperation networks, and performs well in systematic mapping of academic relationships.

In recent years, due to their high medicinal and research value, *Dendrobium officinale* and other medicinal plants of the Dendrobium genus have been the subject of increasingly in-depth research, and the number of related publications has continued to grow. To thoroughly investigate the bioactivity of *Dendrobium officinale* and other medicinal plants of the Dendrobium genus, as well as the progress of research on their products and their applications across various fields, we conducted searches in the PubMed and Web of Science databases, as well as the China Clinical Trials Registry. We then performed statistical analysis of these articles using VOSviewer and CiteSpace.

**Figure 1:**
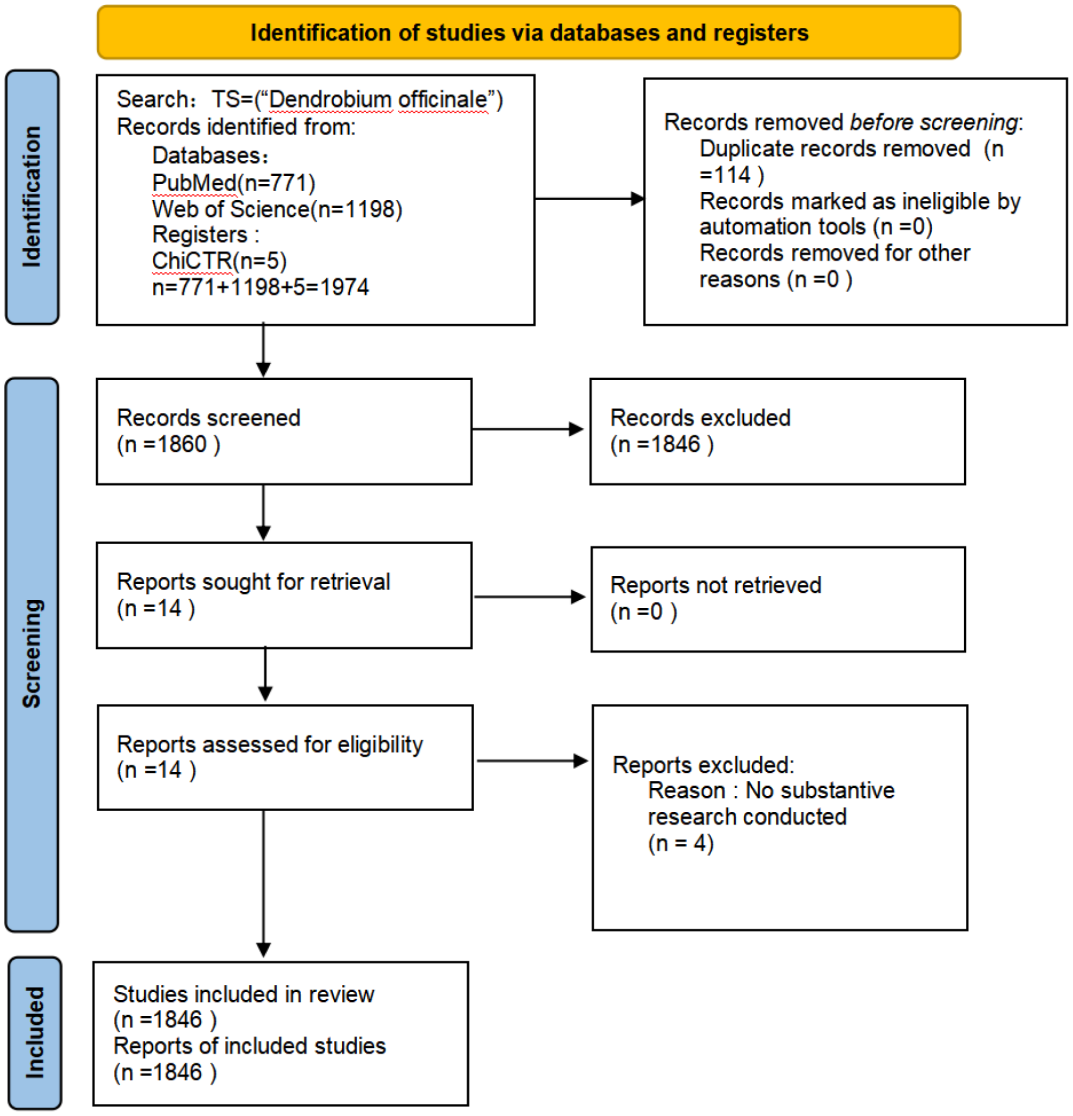
PRISMA flow diagram illustrating the study selection process for *Dendrobium officinale* systematic review. It details the number of records identified (1974), removed (114 duplicates), screened (1860), assessed for eligibility (14), and finally included (1846), with 4 excluded for no substantive research.

This study retrieved data from the PubMed and Web of Science databases as well as the China Clinical Trials Registry. No time restrictions were imposed; articles from all publication years were included. The search strategy “TS=(“*Dendrobium officinale*”)” was employed to conduct a comprehensive search for articles related to *Dendrobium officinale*. We retrieved 771 and 1,198 articles from PubMed and Web of Science, and the China Clinical Trials Registry, yielding 771, 1,198, and 5 articles, respectively. A total of 1,974 articles were initially abstracted. After merging and deduplicating the data, 1,860 articles remained, of which 14 required further screening. After reviewing the full texts of these 14 studies and excluding 4 articles that did not conduct substantive research, 1,846 articles were retained, as shown in **Figure 1**.

### 2.2 Analysis results

#### 2.2.1 VOSviewer

To thoroughly analyze the associative characteristics among keywords, this study employed VOSViewer software to conduct a co-occurrence network analysis of the keywords in the aforementioned 1,846 articles. The analysis method, unit of analysis, and counting method were defined as co-occurrence analysis, all keywords, and full-text counting, respectively. We screened a total of 7,692 keywords and selected 41 keywords with a frequency of 40 or higher. After further removing keywords unrelated to the research content, the final set consisted of 38 keywords. Using VOSViewer, we generated a keyword co-occurrence map. The map is divided into four groups (represented by four different colors), with nodes representing keywords. Lines connecting nodes indicate an association between them, and the thickness of the lines represents the strength of the association. The size of nodes is directly proportional to the occurrence frequency of keywords. This visualization method can vividly reflect the correlation characteristics among keywords and help quickly sort out the internal connections between them.

**Figure 2:**
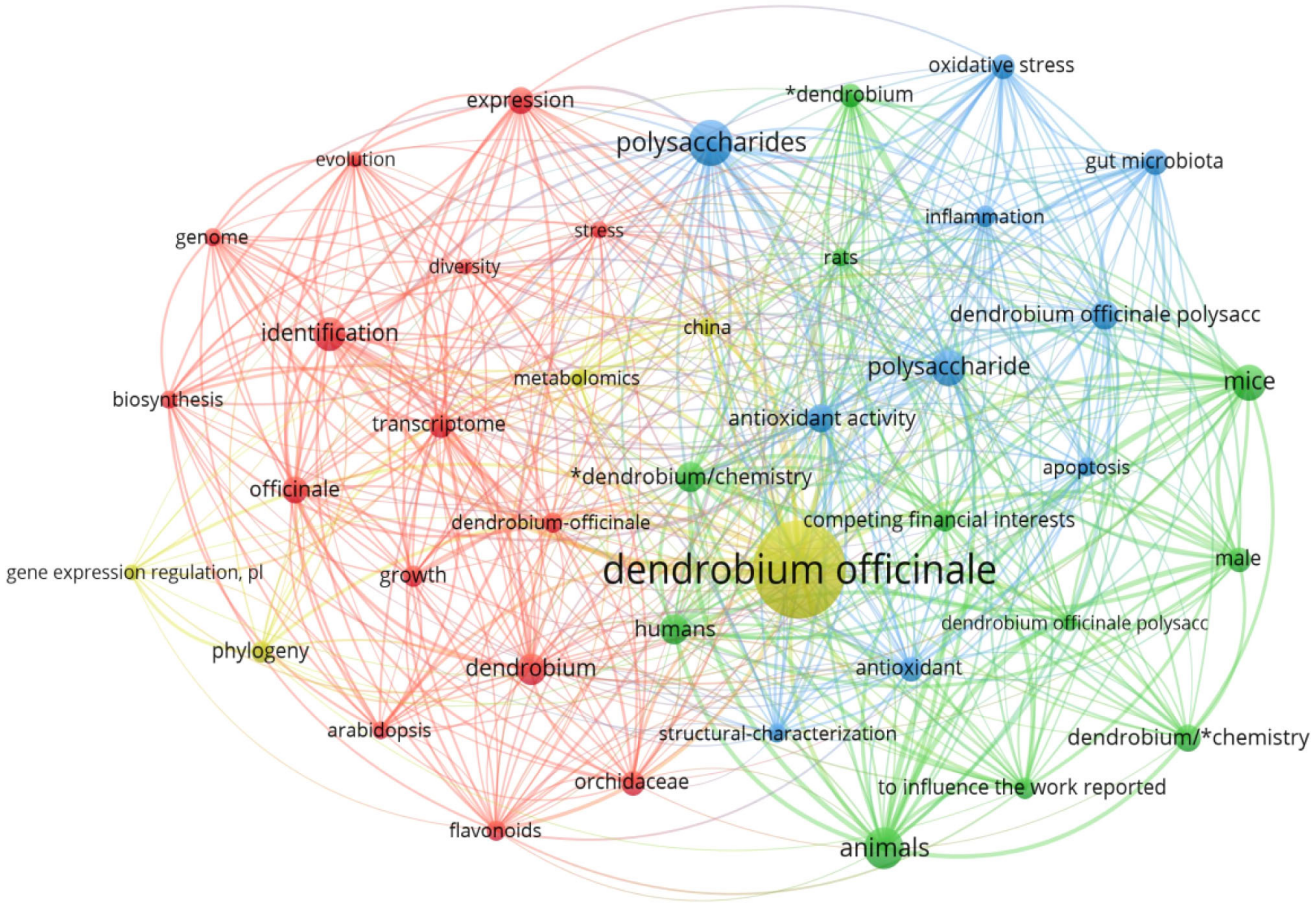
Co-occurrence network visualization map of *Dendrobium officinale* research keywords. Nodes represent keywords, with node size indicating occurrence frequency; colors distinguish research clusters (e.g., red for genetic research, green for animal experiments, blue for pharmacological activity). Core keywords include polysaccharides, oxidative stress, inflammation, and gut microbiota.

Based on the keyword co-occurrence network analysis (**Figure 2**), the following insights can be drawn. First, polysaccharides are the largest and most connected node, confirming their central role in *Dendrobium* research. Second, oxidative stress and inflammation are the most widely connected bioactivity-related keywords, indicating that antioxidant and anti-inflammatory effects are the primary activities attributed to *Dendrobium* polysaccharides. Third, bibenzyls and alkaloids appear as smaller nodes with weak connections to the polysaccharide node, revealing a critical research gap: the synergistic effects between polysaccharides and small-molecule metabolites remain largely unexplored. Finally, the centrally located gut microbiota node with strong connections indicates. As shown in **Figure 2**, the keywords with the highest frequency are Dendrobium plants, animals, humans, and polysaccharides. This indicates that the primary focus of research is on the effects of Dendrobium medicinal plants, as traditional Chinese herbal medicines, on humans and animals. In addition, key terms such as benzyl derivatives, polysaccharides and alkaloids also appear frequently, indicating that such plant extracts serve as the primary research objects in studies related to *Dendrobium officinale* and medicinal Dendrobium species within the Dendrobium genus. This review will therefore use these keywords as a starting point to discuss their biological activities and recent research progress on related products.

#### 2.2.2 Citespace

**Figure 3:**
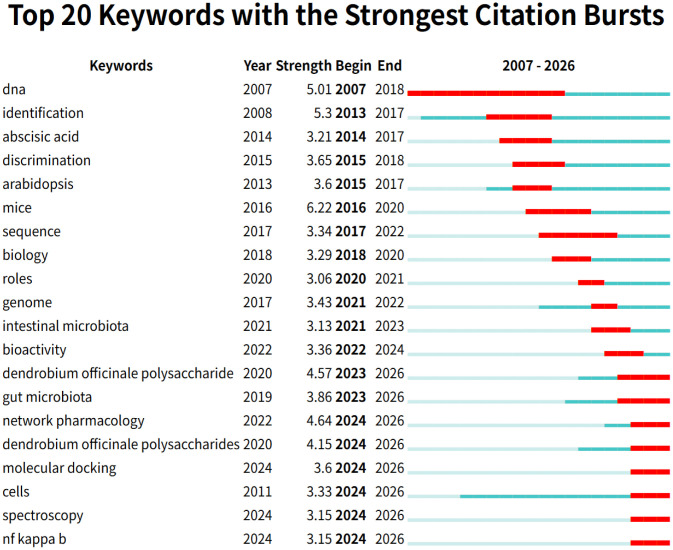
Bar chart showing the top 20 keywords with the strongest citation bursts in *Dendrobium officinale* research from 2007 to 2026. Red segments indicate burst periods, blue segments non-burst periods. Key burst keywords include *Dendrobium officinale* polysaccharide, network pharmacology, and gut microbiota, reflecting recent research trends.

To gain a deeper understanding of the interrelationships among keywords, research hotspots in the field, and evolutionary trends, this study utilized CiteSpace software to perform co-occurrence network and clustering analyses on the aforementioned keywords. Using literature from 2007 to 2026 with “*Dendrobium officinale*” as the theme, and employing the “g-index” for node selection, a keyword highlight map was constructed, as shown in **Figure 2**. “Keyword” refers to the keyword; “Year” indicates the key year when the keyword first garnered high attention or when related research began; “Strength” is a metric in CiteSpace that measures the “explosive growth rate” of a keyword’s citation frequency. The higher the value, the greater the research intensity during that phase. The evaluation criteria are as follows: High emergence strength (≥4.0): represents a “core hotspot” during that phase, with extremely high academic attention; Moderate emergence strength (3.5–4.0): Represents “significant hotspots,” which are key areas for follow-up within the field; Low emergence strength (3.0–3.5): Represents “potential hotspots,” where attention is steadily increasing but has not yet resulted in explosive growth, as shown in **Figure 3**. “Begin” and “End” indicate the start and end years of the keyword’s citation surge period. The segments on the timeline axis on the right side of the image consist of dark and light blocks. The dark blocks correspond to the emergence cycles shown in the “Begin-End” column on the left, representing the phase where research interest is concentrated and citation frequency experiences explosive growth. The light blocks represent non-explosive periods (including the preparatory phase before the surge and the cooling-off phase after the surge). The continuous length of the segments visually reflects the sustained nature of the keyword’s popularity.

We selected 20 of these keywords for in-depth analysis. Research on the bioactivity and products of *Dendrobium officinale* and other medicinal plants in the Dendrobium genus reveals a clear evolutionary trajectory: Early Phase (2007–2020): Centered on keywords such as “DNA,” “identification,” and “discrimination,” this phase focused on species identification and raw material authenticity. At the same time, model organisms such as “mice” and “Arabidopsis” were used to conduct preliminary validation of basic biological characteristics and potential bioactivities. Among these, “mice” emerged as the core model for *in vivo* pharmacological validation during this phase, with the highest emergence intensity of 6.22; The middle phase (2021–2023) gradually shifted toward core components and emerging directions. The surge in keywords such as “intestinal microbiota,” “bioactivity,” and “genome” signaled a research focus on bioactivity validation and the interdisciplinary field of “traditional Chinese medicine–gut microbiota,” laying the foundation for mechanism elucidation; the current phase (2024–2026) has entered a stage of refinement and multi-technology integration, *Dendrobium officinale* polysaccharides (with emergence intensities of 4.57 and 4.15) have become the absolute core. Research has expanded from single-component studies to the synergistic effects of multiple components. Technologies such as network pharmacology and molecular docking have surged in tandem with the NF-κB pathway, cells, and spectroscopy, establishing a complete technical chain of “component separation – target prediction - molecular validation - bioactivity evaluation,” providing mechanistic support and technical assurance for product R&D. It is worth noting that keywords related to genetic engineering, gene editing, and multi-omics integration do not appear in the burst list. This may reflect that these technologies are still in early stages of application in *Dendrobium* research. Future efforts could usefully explore their potential to address current mechanistic and quality control challenges.

To further investigate the development history and trends of *Dendrobium officinale* products, we screened 103 product-related articles from the literature and selected 21 of them to construct a keyword visualization map.

**Figure 4:**
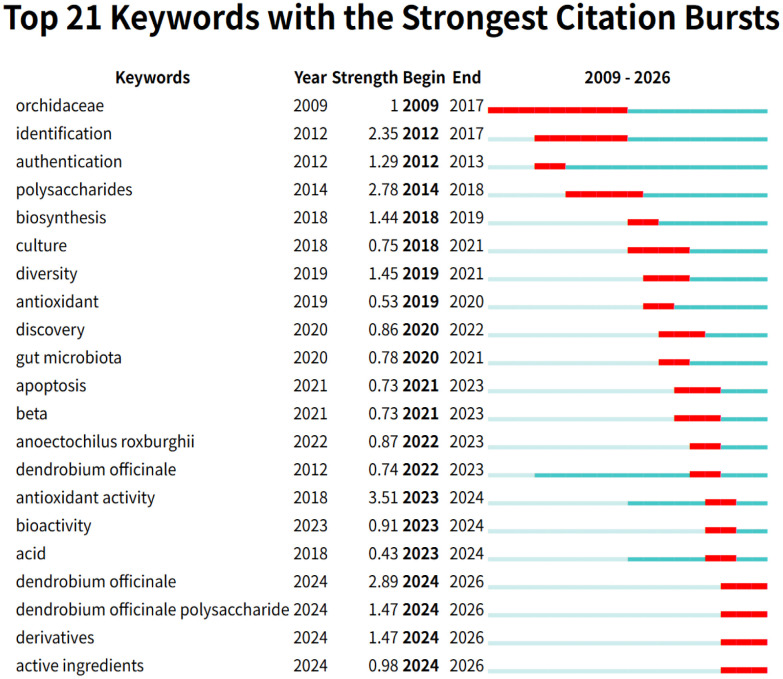
Citation burst timeline chart of the top 21 keywords in *Dendrobium officinale* product research (2009–2026). Red bars indicate burst periods, light blue bars non-burst periods. Key application hotspots include *Dendrobium officinale*, its polysaccharides, and active ingredients (2024–2026).

This indicates that research on *Dendrobium officinale* products exhibits a pattern of “strong bioactivity but weak product development”: early studies focused on basic research such as the Orchidaceae family, species identification, and authentication, and gradually expanded to include preliminary investigations into active components such as polysaccharides, as well as initial explorations of bioactivities related to antioxidant effects and gut microbiota; In the mid-phase (2022–2023), the focus shifted to the validation of biological activities. Antioxidant activity emerged as the core research hotspot with the highest intensity score of 3.51. The keyword “*Dendrobium officinale*” regained prominence, and research expanded to include closely related medicinal plants such as Anoectochilus roxburghii, significantly broadening the scope and specificity of activity studies; Currently (2024–2026), the field has entered the stage of precise deepening of active ingredient research and the initial phase of product commercialization. *Dendrobium officinale* and its polysaccharides remain the core research subjects, Research on active ingredients has become increasingly refined, while “derivatives”—as the only keyword directly linked to product development—has emerged for the first time. However, the overall number of product-oriented keywords remains scarce, and their emergence intensity (1.47 or lower) is far below that of bioactivity-related keywords. This highlights that the field is currently still dominated by the exploration of biological activity and mechanism analysis, with the transition toward product commercialization still in its infancy. In summary, **Figure 4** offers two main insights for future product development. First, the gap between bioactivity research and product commercialization is substantial, as evidenced by the low burst strength of product-related keywords. Second, the emergence of “derivatives” signals an early but promising shift toward commercialization. Future efforts should therefore prioritize formulation development, quality control, and clinical translation.

## 3 Resource distribution

In China, Dendrobium species are primarily found in regions south of the Yangtze River, forming two major industrial belts centered on Yunnan and Zhejiang (Yang et al., 2020; Li et al., 2026).Yunnan, as the wild origin region, benefits from the climatic advantages of low latitude and high altitude, resulting in high yields and low cultivation costs; Zhejiang, on the other hand, was the first to achieve artificial cultivation and industrialization, innovating cultivation models such as greenhouse semi-wild cultivation and epiphytic cultivation on living trees. At the same time, the demand for health supplements in the Jiangsu-Zhejiang region has significantly driven the development of the *Dendrobium officinale* industry.

The cultivation methods for *Dendrobium officinale* are primarily categorized into Understory Cultivation (UC), Greenhouse Cultivation (GC), and Semi-Wild Cultivation (SWC). These cultivation methods, together with the geographical environment, jointly influence the distribution of *Dendrobium officinale* resources. *Dendrobium officinale* from different cultivation methods and origins exhibits specific biomarkers: catechins and eugenic acid in the UC method; eugenol and caffeic acid in the GC method; quercetin in the SWC method; and 3,5 -dihydroxybenzoic acid from Zhangzhou, Fujian, and dihydroyangmeoside from Chishui, Guizhou, among others, can serve as key indicators for distinguishing origin and cultivation mode (Yuan et al., 2020). In addition, there are significant differences in the volatile components, methanol extracts, polysaccharides and other components of *Dendrobium officinale* under different cultivation modes (Ma et al., 2018; Yang et al., 2020; Sun et al., 2024).

In terms of cultivation, different cultivation methods—including greenhouse cultivation, understory planting, and semi-wild cultivation—can affect the chemical composition and bioactive properties of *Dendrobium officinale*. Among these, the understory cultivation method exhibits superior flavonoid content, antioxidant activity, and α-glucosidase inhibitory activity. Standardized cultivation systems further improve the uniformity and stability of bioactive compounds by unifying key technical parameters such as light intensity, temperature, humidity, ventilation, substrate composition, fertilization regime, irrigation frequency, and harvesting period. By eliminating environmental fluctuations and inconsistent management practices, standardized cultivation significantly reduces batch-to-batch variations in polysaccharides, alkaloids, bibenzyls, flavonoids and other key metabolites. Uniform growth conditions promote coordinated expression of genes involved in the biosynthesis of active components, thereby enhancing the overall quality consistency and pharmacological stability of medicinal Dendrobium materials (Li et al., 2026).Different producing areas and cultivation patterns can also promote the formation of specific chemical markers. In addition, relevant metabolomic studies have confirmed that metabolic pathways such as aminobenzoic acid degradation and phenylpropanoid biosynthesis serve as the core regulatory mechanisms underlying such compositional differences, which lays a theoretical foundation for the cultivation improvement, quality control and product research and development of *Dendrobium officinale* (He et al., 2025). In addition, under the same cultivation conditions, different varieties of *Dendrobium officinale* exhibit distinct physiological responses to low-temperature stress, and such differences exert certain effects on their growth status and the accumulation of active components. A low-nitrogen environment also exerts a positive effect on the formation of active ingredients in Dendrobium (Wu et al., 2023a). In reagent production, variety selection should be integrated with habitats and cultivation modes to improve the quality and yield of *Dendrobium officinale* (Zhang et al., 2020).

## 4 Major medicinal components

*Dendrobium officinale* contains abundant and diverse metabolites. It should be emphasized that this medicinal herb harbors hundreds of metabolites in total. Its comprehensive pharmacological effects arise from the joint action of multiple components rather than a single substance. Such a multi-component system features complementary efficacy, lowered safety risks and more stable pharmacological performance, which constitutes a prominent advantage of herbal medicines.

To clarify the functional characteristics and action rules of each constituent in depth, we elaborate the biological activities of major bioactive components separately in the following sections. This individual analysis helps reveal the contribution of each substance, and further lays a foundation for interpreting their synergistic mechanisms.

### 4.1 Polysaccharides

#### 4.1.1 Structural characteristics

**Figure 5:**
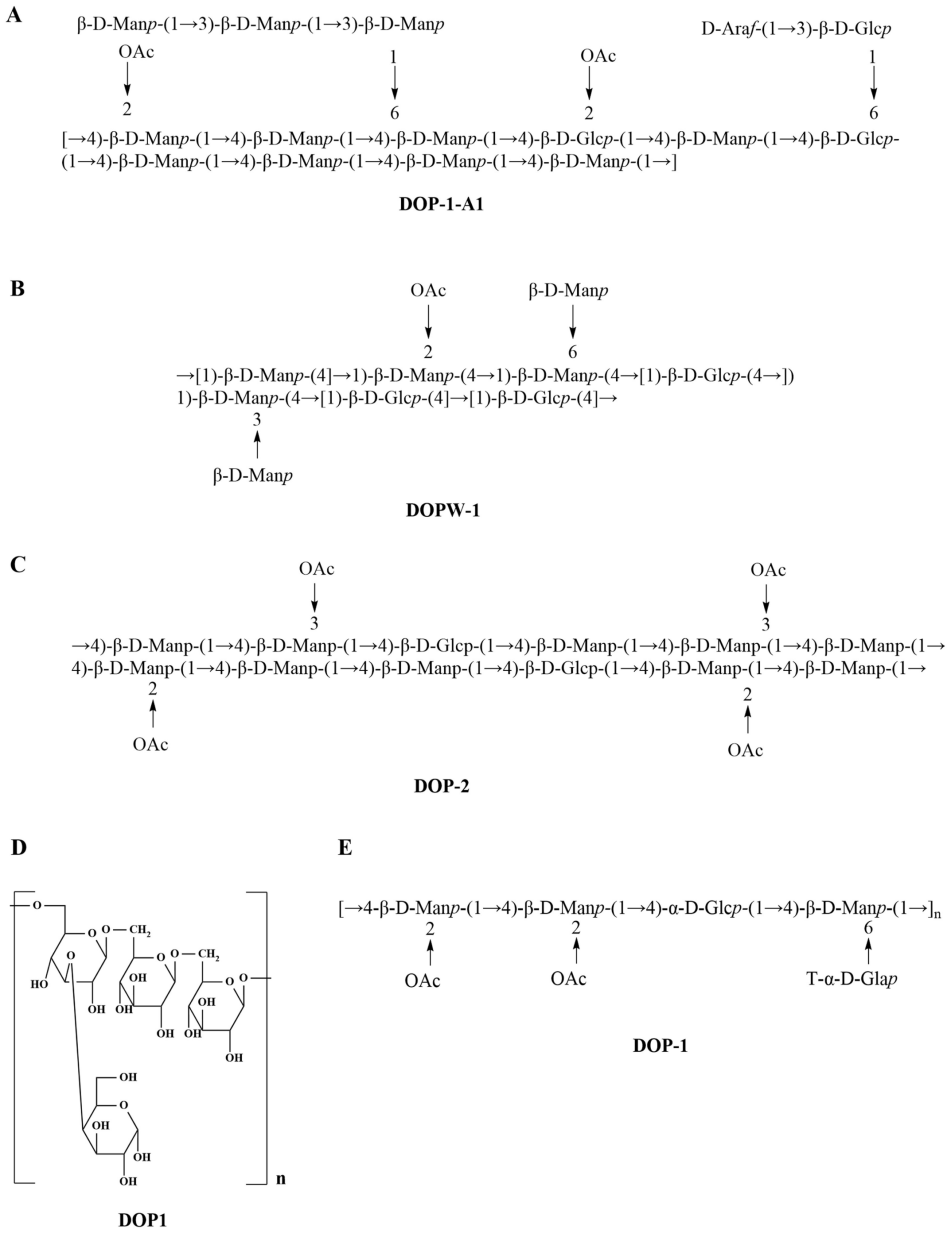
Molecular formula of Dendrobium polysaccharides. Schematic illustration of five polysaccharide structures labeled **(A)** through **(E)**. Panels **(A–C)** show linear and branched glycosidic linkage diagrams with specific acetylation (OAc) and linkage positions. Panel **(D)** presents a chemical structure diagram for DOPI, displaying a repeating mannopyranose backbone. Panel **(E)** displays a summarized polysaccharide repeat unit with acetylation sites and a terminal a-D-Glap group. Each panel includes the name of the polysaccharide variant (Liang et al., 2025).

*Dendrobium officinale* polysaccharides (DOP) are one of its primary active components, consisting mainly of mannose, glucose, xylose, arabinose, and galactose (Gong et al., 2025). In terms of structural characteristics, the main chain consists of β-1,4-linked mannose residues, while the side chains contain α-1,6-linked glucose residues; the molecular weight distribution ranges from 10 to 200 kDa (Tian, 2020; Huang et al., 2022) **Figure 5** illustrates the chemical structures of five representative *Dendrobium* polysaccharide fragments (Liang et al., 2025).

Structure-activity relationship studies indicate that branching degree is positively correlated with immunological activity. Polysaccharides with branching degree over 0.3 greatly improve phagocytic capacity, while linear polysaccharides have much lower activity. Molecular weight determines their acting sites: polysaccharides above 100 kDa target immune cell surface receptors, and those below 50 kDa enter cells to regulate PI3K/Akt signaling. High-molecular-weight Dendrobium polysaccharides exhibit better biological activity (Zha et al., 2013). Ultrasonication combined with hydrogen peroxide degradation of crude polysaccharides from *Dendrobium officinale* yields four polysaccharide fractions with different molecular weights. The polysaccharide with the lowest molecular weight exhibits the weakest immunomodulatory capacity, likely due to its small molecular weight, short chain length, and fewer active sites for immunomodulation (Zhao et al., 2023b; Luo et al., 2024).

##### 4.1.2 Extraction process

In recent years, the extraction technology of *Dendrobium officinale* Polysaccharides (DOP) has been continuously improved and developed, with different processes exhibiting significant differences in yield, structural retention, and biological activity. As a conventional extraction method, hot-water extraction presents an extraction yield of 15%–25%. The obtained polysaccharides have a molecular weight of 50–200 kDa, with their backbone mainly composed of β-1,4-mannose. These polysaccharides exhibit prominent immunomodulatory activity; however, high temperatures can easily lead to the degradation of antioxidant groups. With the assistance of cavitation effect, the ultrasound-assisted extraction method can reduce the treatment time to 30–60 minutes and increase the extraction rate to 20%–30%.The proportion of low-molecular-weight components (<50 kDa) in the product rises, and the content of glucose and galactose increases. The DPPH radical scavenging rate is 15% higher than that of traditional methods, but immunological activity decreases slightly. Enzymatic extraction (cellulase + pectinase) specifically degrades cell walls, resulting in a concentrated molecular weight range (20–100 kDa) in the product. The proportions of arabinose and xylose in the monosaccharide composition increase significantly, and its anti-inflammatory activity—with an inhibition rate of up to 40% against TNF-α—is particularly notable, rendering it suitable for the development of anti-inflammatory drugs. Microwave-assisted extraction takes only 5–15 minutes, with a high extraction yield of 25%–35%. Polysaccharides isolated by this method possess immunomodulatory and antioxidant capacities, which can promote the secretion of IL-2 and enhance SOD activity; however, power must be strictly controlled to prevent carbonization. Supercritical fluid extraction (SFE) uses CO_2_ as the solvent. The resulting product demonstrates high purity and structural integrity (with a molecular weight ranging from 5 to 50 kDa) and exhibits significant antitumor activity, as evidenced by a 25% increase in lung cancer cell apoptosis. However, its yield is relatively low, ranging from 10% to 18%; this yield can be increased to 22% by adding an ethanol entrainer. Pulsed electric field (PEF) is a cutting-edge technology that can release intracellular polysaccharides within milliseconds, achieving yields of 28–32% with intact product structure (50–80 kDa) and a 35% increase in retinal cell protective activity; however, equipment costs limit its large-scale application. Notably, combined processes—such as the integration of ultrasonic and enzymatic methods—can further optimize extraction efficiency and product activity through synergistic effects, increasing the yield to 35% and improving antioxidant activity by 30% (Ning et al., 2019; Yu et al., 2021; Zhao, 2022)Several advanced extraction technologies have been developed to further enhance the yield and bioactivity of Dendrobium polysaccharides, including deep eutectic solvent extraction (DES), pulsed electric field (PEF), subcritical water extraction, and fermentation-assisted extraction. These green and efficient techniques can significantly increase polysaccharide yield, better retain molecular structure and branching degree, enhance immunomodulatory, antioxidant and eye-protective activities, and reduce energy consumption and environmental pollution, making them suitable for large-scale industrial production. **Table 1** summarizes the effects of different extraction methods on the yield and physicochemical properties of polysaccharides from Dendrobium species.

**Table 1:** Effect of different extraction methods on dendrobium polysaccharide yield and characteristics.

Extraction method	Yield	Time	Major bioactivity product characteristics	DOP molecular weight & structural features	Applicable scenarios
Hot water extraction method	Moderate (15-25%)	Long (2–4 hours)	Prominent immunomodulatory activityHigh molecular weight, strong immunogenicity	50–200 kDa Backbone mainly composed of β-1,4-mannose	Traditional pharmaceuticals, health supplements
Ultrasound-assisted	Medium-high (20%-30%)	Short (< 1h)	DPPH scavenging rate 15%; slight decrease in immune activityOutstanding antioxidant properties, low cost	Increased proportion of <50 kDa components Increased glucose and galactose content	Functional foods, antioxidants
Enzymatic method	Medium (18-28%)	Medium (1–2 hours)	Notable anti-inflammatory activity (TNF-α inhibition rate ↑40%)Structure is uniform, with significant anti-inflammatory activity	20–100 kDa Significantly increased proportion of arabinose and xylose	Anti-inflammatory drugs, high-end cosmetics
Microwave-assisted	High (25%-35%)	Extremely short (< 15m)	Immunomodulatory (promotes IL-2 secretion); antioxidant (enhances SOD activity)Dual-active, suitable for industrialization	Not specified	Fast-moving consumer goods, compound preparations
Supercritical Fluid	Low (10-18%)	Medium (1–2 hours)	Significant antitumor activity High purity, potent antitumor activity	5–50 kDa High purity; intact structural integrity	Pharmaceutical raw materials, injectable-grade polysaccharides
High-voltage pulsed electric field	High (28%-32%)	Extremely short (millisecond level)	Retinal cell protective activityStructurally sound with outstanding eye-protecting properties	50–80 kDa, intact structure	Premium pharmaceuticals, eye health products

This table summarizes the yield, processing time, product characteristics, and applicable scenarios of *Dendrobium* polysaccharides obtained via different extraction methods, based on data from previously published studies. All data were extracted and collated from peer-reviewed literature.

##### 4.1.3 Biological activity

**Figure 6:**
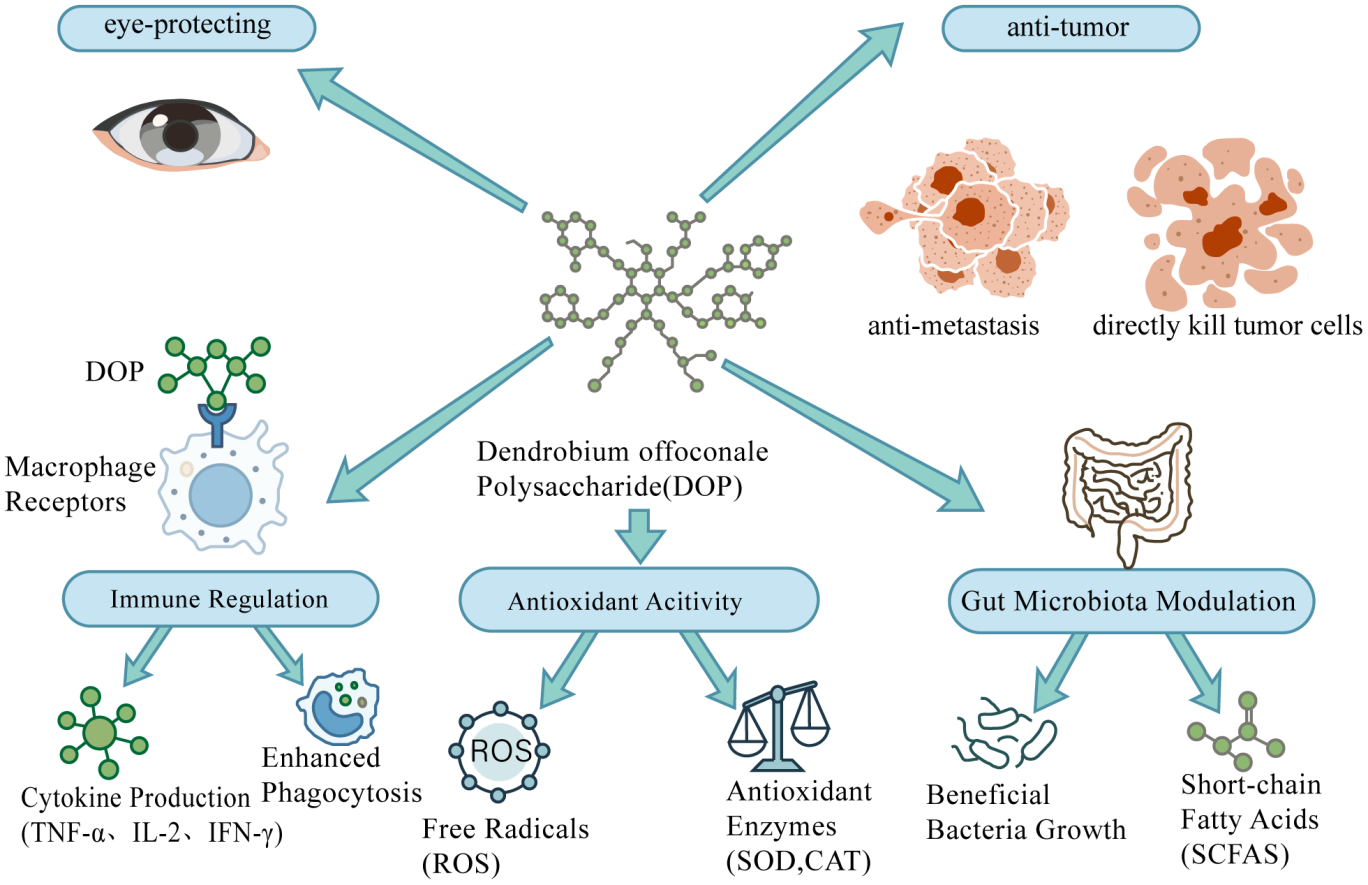
Schematic diagram illustrating the core bioactivities and mechanisms of *Dendrobium officinale* Polysaccharide (DOP). It depicts five key functional pathways: eye protection, anti-tumor activity, immune regulation, antioxidant activity, and gut microbiota modulation, leading to improved ocular health, suppressed tumor growth, enhanced immunity, reduced oxidative stress, and optimized gut homeostasis.

**Figure 6** schematically illustrates the core bioactivities and underlying mechanisms of *Dendrobium officinale* polysaccharide (DOP).

###### 4.1.3.1 Immunomodulatory effects

*Dendrobium officinale* polysaccharides can effectively enhance the overall immune function of the body, and their mechanism of action is mainly divided into two major aspects. First, in terms of immune cell regulation, *Dendrobium officinale* polysaccharides promote the proliferation and differentiation of T lymphocytes and elevate the CD4^+^/CD8^+^ ratio. Meanwhile, they strengthen the phagocytic and antigen-presenting capacities of macrophages, stimulate natural killer (NK) cells to enhance anti-tumor effects, and accelerate the proliferation of B lymphocytes to facilitate the synthesis of more antibodies, thereby exerting immunomodulatory and immunoenhancing effects. Second, with regard to cytokine regulation, these polysaccharides modulate the expression of pro-inflammatory factors such as IL−2, IFN−γ and TNF−α, inhibit the secretion of anti-inflammatory factors including IL−4 and IL−10, and further regulate the activation of the NF−κB signaling pathway (Guo et al., 2024).Its activation of the complement system manifests as activation of the classical complement pathway, enhancement of complement C3 and C4 activity, and promotion of immune complex clearance. Additionally, it exerts significant effects on immune organs, which includes promoting the development of the thymus and spleen, increasing the number of immune cells in lymph nodes, and improving the microenvironment of immune organs.

###### 4.1.3.2 Antioxidant effects

Extracts of *Dendrobium officinale*, such as polysaccharides and alkaloids, exhibit significant antioxidant activity. Their mechanism of action primarily involves four aspects: First, their ability to directly scavenge free radicals, including hydroxyl radicals (·OH), superoxide anions (O_2_^-^), and DPPH radicals, as well as their ability to inhibit lipid peroxidation (Yao et al., 2019).Second, they can enhance the antioxidant enzyme system, which is reflected in increased superoxide dismutase (SOD) activity, upregulated catalase (CAT) expression, and elevated glutathione peroxidase (GSH-Px) levels. These changes collectively help maintain the intracellular redox balance (Qiu, 2017). Third, it exerts regulatory effects on signaling cascades associated with oxidative stress. To be specific, it triggers the activation of the Nrf2/HO-1 axis, blocks the MAPK signaling cascade, modulates PI3K/Akt pathway activity, as well as preserves the regular physiological performance of mitochondria. Finally, it delivers potent cell-protective properties. It can alleviate oxidative DNA injury, safeguard mitochondrial performance, keep the cell membrane intact, and slow down the progression of cellular aging.

###### 4.1.3.3Eye protection and vision improvement

Modern pharmacological studies have confirmed that *Dendrobium officinale* possesses multiple mechanisms for eye protection (Xiao and Chen, 2020). Its neuroprotective effects primarily manifest as protection of retinal ganglion cells, reduction of excitotoxicity, promotion of neurotrophic factor expression, and maintenance of optic nerve axon integrity. Regarding the circulatory system, it improves ocular microcirculation, promotes retinal blood flow, maintains the integrity of the blood-retinal barrier, and inhibits abnormal neovascularization. It provides antioxidant protection via clearing free radicals in retinal tissues, defending visual cone and rod cells, relieving light-induced damage and slowing the progression of lens clouding. For metabolic modulation, it optimizes retinal glucose metabolism, stabilizes the function of retinal pigment epithelial cells, balances the ocular immune microenvironment, and boosts tear production (Li et al., 2016, Li et al., 2025a).

###### 4.1.3.4 Antitumor activity

Accumulating research evidence indicates that *Dendrobium officinale* can markedly suppress a wide range of tumor cell lines, such as lung, gastric, colorectal and breast cancer cells. Its underlying pharmacological mechanisms are summarized as follows:

####### 4.1.3.4.1 Direct antitumor effect

Dendrobium polysaccharides exert direct antitumor effects by regulating multiple cellular signaling pathways. A review study (Guo et al., 2024) indicates that these polysaccharides primarily exert their antitumor effects by activating apoptosis-related pathways, regulating key cell cycle proteins, and inhibiting the PI3K/Akt and MAPK signaling pathways. Specifically, they induce apoptosis in tumor cells and arrest cells in the G0/G1 phase, thereby suppressing abnormal cell proliferation. In colon cancer cells, *Dendrobium officinale* polysaccharides can induce mitochondrial dysfunction via the ROS-AMPK-autophagy pathway, leading to excessive autophagy and cell death, thereby further elucidating its antitumor molecular mechanisms (Zhang et al., 2021).

####### 4.1.3.4.2 Anti-metastatic effects

*Dendrobium officinale* polysaccharides (DOP) exert a significant inhibitory effect on the migration and invasion of tumor cells. Accumulating studies have demonstrated that their functional effects are exerted through multiple regulatory mechanisms.

Suppression of epithelial-mesenchymal transition (EMT): DOP restrains the expression of EMT-associated proteins including N-cadherin and vimentin, and meanwhile upregulates E-cadherin. This mechanism hinders the mesenchymal transformation of tumor cells and impairs their migratory capacity (Zhao et al., 2023a).

Regulation of the matrix metalloproteinase (MMP) system: DOP inhibits the expression and activity of matrix metalloproteinases MMP-2 and MMP-9, while increasing the levels of their tissue inhibitors TIMP-1 and TIMP-2, thereby reducing extracellular matrix degradation and impeding tumor cell invasion (Xu et al., 2022).

Inhibition of the Rho/ROCK signaling pathway: DOP reduces the motility of tumor cells by inhibiting the Rho/ROCK signaling pathway, thereby further suppressing their metastatic potential (Xu et al., 2022).

These mechanisms of action work synergistically to effectively reduce the invasive and metastatic capabilities of tumor cells.

####### 4.1.3.4.3 Immunomodulatory effects

*Dendrobium officinale* polysaccharides play a significant role in enhancing the body’s antitumor immune response. In terms of cellular immunity, they promote the differentiation of T lymphocytes into Th1 cells, increase the secretion of cytokines such as IFN-γ, TNF-α, and IL-2, and enhance the cytotoxic activity of CD8+ cytotoxic T lymphocytes (CTLs) (Zhao et al., 2023b),thereby enhancing the killing of tumor cells.

###### 4.1.3.5 Effects on gut microbiota

*Dendrobium officinale* polysaccharides are primarily metabolized by the gut microbiota in the large intestine. Metabolic decomposition generates oligosaccharides and short-chain fatty acids (SCFAs). These bioactive substances regulate the composition and diversity of intestinal flora, so as to improve human physical health. Overconsumption of calories may disrupt serum levels of total cholesterol (TC), triglycerides (TG), low-density lipoprotein cholesterol (LDL-C), and high-density lipoprotein cholesterol (HDL-C),further contributing to metabolic disorders including obesity and type 2 diabetes mellitus (Ding et al., 2019; Xue et al., 2019). These metabolic issues are associated with various diseases and may even induce cancer and cognitive impairment. Research has demonstrated that *Dendrobium officinale* polysaccharides are capable of decreasing fasting blood glucose (FBG) levels, ameliorating insulin resistance, reducing concentrations of TC, TG and LDL-C, while at the same time elevating HDL-C levels. while simultaneously increasing HDL-C levels. Their potential mechanisms of action may include: (1) bypassing digestion in the stomach and small intestine, where they are utilized by the gut microbiota to produce short-chain fatty acids and other substances that regulate the gut microbiota composition (Fu et al., 2019; Li et al., 2019a; Fan et al., 2021; Wu et al., 2022); (2) Regulating lipid metabolism and cholesterol homeostasis, thereby reducing cholesterol absorption and utilization (Qu et al., 2021);(3) Influencing glucose metabolism by reducing the activity of glucose-metabolizing enzymes and regulating intestinal function and hormone secretion (Wu et al., 2023b)); (4) Regulating protein metabolism, increasing taurine levels, and inhibiting the elevation of branched-chain amino acids caused by insulin resistance (Wu et al., 2023b).

Mitochondria are critical for sustaining cellular metabolic balance. Impaired mitochondrial function serves as a typical pathological basis for diabetes and its associated complications. In C57BL/6J mice, the potential cellular mechanisms by which *Dendrobium officinale* polysaccharides regulate host metabolism include: upregulating uncoupling protein 1 (UCP1) in mitochondria, activating peroxisome proliferator-activated receptor gamma coactivator 1-alpha (PGC-1α) in brown adipose tissue, regulating thermogenesis and the expenditure of excess energy, enhancing insulin signaling and hepatic glycogen synthesis, and improving glucose homeostasis (Wu et al., 2023b). *Dendrobium officinale* polysaccharides can also alleviate mitochondrial dysfunction by enhancing the activity of mitochondrial respiratory chain complexes, upregulating phosphorylated AMP-activated protein kinase (p-AMPK)/AMPK, and improving the function of the tricarboxylic acid cycle (Chen et al., 2023b). Furthermore, *Dendrobium officinale* polysaccharides intervene in glycogen synthesis and glucose metabolism by activating the PI3K/Akt pathway (Wang et al., 2018); they mediate the PKA and Akt/FoxO1 pathways to further promote hepatic glycogen synthesis, inhibit glycogenolysis, and suppress hepatic gluconeogenesis (Liu et al., 2020b);and they enhance lipid metabolism by regulating peroxisome proliferator-activated receptors (PPARs) (Qu et al., 2021).

The biological activity of *Dendrobium officinale* polysaccharides is positively correlated with their structure. Their molecular weight is positively correlated with hypoglycemic and hypolipidemic effects; the degree of acetylation is negatively correlated with hepatoprotective and nephroprotective effects and influences serum compound metabolism; and the ratio of mannose to glucose has varying effects on different metabolic pathways (Wu et al., 2023b).

### 4.2 Alkaloids

#### 4.2.1 Structural characteristics

**Figure 7:**
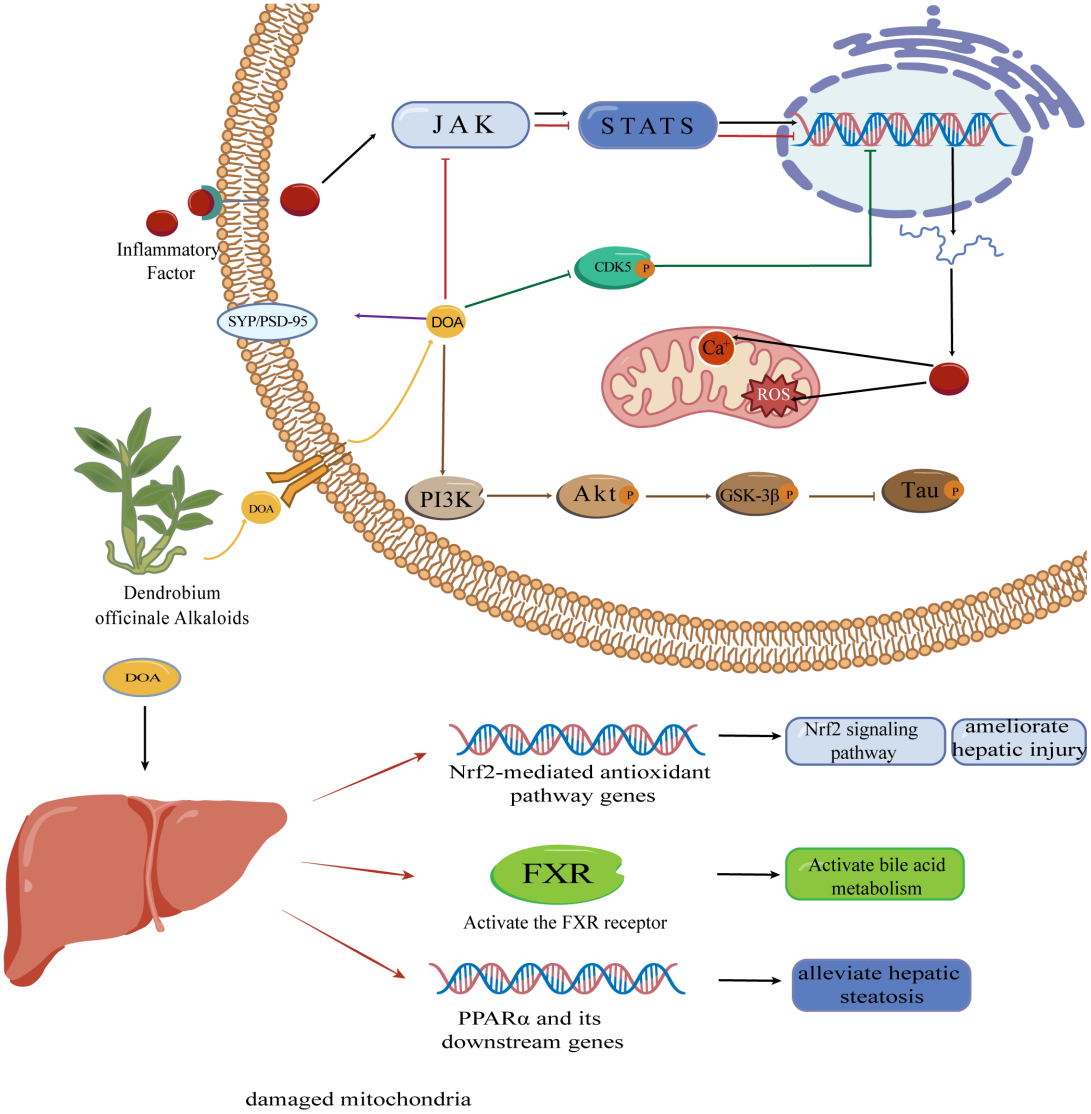
Schematic diagram illustrating the core neuroprotective and hepatoprotective mechanisms of *Dendrobium officinale* Alkaloids (DOA). It depicts multiple key pathways: neuroinflammation inhibition, mitochondrial quality control, Nrf2-mediated antioxidant signaling, PPARα-regulated lipid metabolism, and mitophagy activation, leading to reduced neuroinflammation, enhanced mitochondrial function, ameliorated hepatic injury, alleviated hepatic steatosis, and improved cellular homeostasis.

Dendrobium alkaloids are a class of nitrogen-containing compounds with unique ring structures. It owns a fused tetracyclic structure, which generally contains pyrrolidine or piperidine ring moieties. To date, more than 100 alkaloids have been isolated and identified from plants of the Dendrobium genus. These primarily include imidazoline alkaloids (such as the Dendrobine A, B, and C series, which possess characteristic imidazoline ring structures (Gao et al., 2023))), indole alkaloids (such as Dendrobin, which possesses a unique indole skeleton and often forms complexes with polysaccharides (Tian, 2020; Wang et al., 2025b)))),and other types (including dibenzylisoquinolines, diphenylheptanes, phenyl-ethylamines, etc.).The chemical structures of Dendrobium alkaloids are shown in **Figure 7**.

#### 4.2.2 Extraction methods

Alkaloids are core bioactive components of Dendrobium species. Various extraction methods have been established for total alkaloid preparation (Mou et al., 2021). For traditional techniques, acid solvent extraction is most commonly applied, including acidic ethanol extraction and acidified water maceration, which effectively enhance alkaloid solubility and release.

In terms of green extraction technologies, ultrasound-assisted extraction and supercritical fluid extraction (SFE) have been widely utilized (Mou et al., 2021). In addition, PEG-based solvent extraction and subcritical water extraction have also been developed. Among them, the PEG-200 system optimized by response surface methodology provides highly efficient extraction for alkaloids (Zhang et al., 2016),Notably, advanced synergistic extraction strategies, such as supercritical CO_2_ extraction, ultrasonic-assisted ionic liquid extraction, subcritical fluid extraction, and enzyme-assisted synergistic extraction, can further improve the extraction yield and purity of Dendrobium bioactive components, especially alkaloids. These advanced methods effectively preserve the structural integrity of heat-sensitive alkaloids, thereby improving their neuroprotective, hepatoprotective, and anti-inflammatory activities. Furthermore, such green and efficient extraction techniques meet the requirements of high efficiency, environmental friendliness, and sustainability for industrial production of high-quality *Dendrobium officinale* products.

#### 4.2.3 Biological activities

The alkaloids isolated from Dendrobium exhibit a variety of biological activities, including neuroprotective, hepatoprotective (Xia et al., 2023; Peng et al., 2024), and antidepressant effects (Shi et al., 2025).

Most alkaloids derived from typical medicinal herbs such as *Coptis chinensis* and *Anisodus tanguticus* have simple molecular skeletons, which lead to non-specific target binding and considerable toxic and side effects. In sharp contrast, Dendrobium alkaloids feature a distinctive fused polycyclic structure. This structural characteristic not only enhances their target-binding specificity and minimizes off-target effects, but also endows them with low biological toxicity due to the inherent mild properties of dual-purpose medicinal and edible Dendrobium. Beyond general pharmacological actions of plant alkaloids, Dendrobium alkaloids exert unique pharmacological effects targeting the eyes, cardiovascular system and gastrointestinal tract (Mou et al., 2021; Fan et al., 2023; Oladeji et al., 2024).More importantly, they protect the nervous system and liver through multiple exclusive regulatory pathways, which differ substantially from the conventional action modes of ordinary plant alkaloids. Furthermore, they exert dual protective impacts on nervous system and liver via multiple pathways. **Figure 7** summarizes the general biological mechanisms of plant alkaloids as well as the unique core bioactivities and signaling pathways of *Dendrobium officinale* alkaloids (DOA), highlighting their mechanistic particularities.

##### 4.2.3.1 Neuroprotection

Dendrobium alkaloids deliver neuroprotective effects via multiple regulatory pathways. They primarily inhibit the JAK−STAT signaling cascade induced by oxidative damage, lower the expression of pro-inflammatory and pro-apoptotic factors, relieve intracellular calcium overload and excessive ROS accumulation (Liu et al., 2020a); Inhibiting CDK5 activation and blocking the Aβ_1-42_-induced apoptosis pathway in PC12 cells (Zhang et al., 2023);Upregulating synaptic-associated proteins (SYP/PSD-95) to protect neurons, and reducing Aβ25–35-induced cytotoxicity and synaptic damage (Zhang et al., 2017); Regulating PI3K/Akt/GSK-3β pathway to suppress Tau hyp erphosphorylation, protecting neurons and improving cognitive function.

##### 4.2.3.2 Protection of the liver

Dendrobium alkaloids can alleviate liver damage through various mechanisms: Alkaloids can modulate glucose and lipid-related genes in liver metabolism and enhance the expression of Nrf2 antioxidant pathway genes, exerting a synergistic effect in liver injury through these mechanisms (Xu et al., 2017);By activating PPARα and its downstream genes, they effectively reduce hepatic steatosis, thereby improving pathological changes associated with non-alcoholic fatty liver disease (Xu et al., 2024). Activating Nrf2/PPARγ/SOD2 pathway to inhibit necroptosis, thereby alleviating CCl4-induced acute liver injury (Yang et al., 2025); Protecting against acute liver injury by inducing Nrf2 pathway and enhancing antioxidant activity (Li et al., 2019b); Protecting the liver via modulating hepatic bile acid profile and activating FXR-mediated bile acid metabolism, which restores hepatic lipid homeostasis and ameliorates lipid accumulation and liver steatosis (Huang et al., 2019).

### 4.3 Biphenyl compounds

#### 4.3.1 Structural characteristics

Biphenyl compounds are among the most bioactive chemical constituents in Dendrobium, consisting primarily of a benzylidene core formed by two benzene rings linked via a methylene bridge. Based on this core, the substitution of hydroxyl and methoxy groups at different positions on the benzene rings and at the α-position, along with variations in the number of substituents and polymeric structures, gives rise to different benzylidene derivatives. This also accounts for the differences in physicochemical properties and biological activity among various benzylidene compounds (Meng et al., 2023a, Meng et al., 2023b; Dai et al., 2024). Bibenzyl compounds can be classified into simple bibenzyl and dibenzyl types. Typical simple bibenzyl compounds include 1,2-dihydroxy-3-methoxydibenzyl isolated from Dendrobium moniliforme, dendrobin from Dendrobium nobile, maolansu from Dendrobium malleatum, as well as 3,4’,5-trihydroxy-3’-methoxybibenzyl derived from Dendrobium purpureum (Zheng et al., 2018; Wu et al., 2020; Chen et al., 2022a; Zhang et al., 2025).Bibenzyl derivatives, such as seven novel bibenzyl dimers (fimbriadimerbibenzyls A–G) from Dendrobium fimbriatum (Xu et al., 2014; Cakova et al., 2017),and the dibenzyl compound Dendrobin A from Dendrobium chrysanthum (Yu et al., 2024), among others.

#### 4.3.2 Extraction methods

Biphenyl constituents are commonly isolated through multiple technical approaches, including ethanol soaking, ultrasonic-assisted extraction, chromatographic fractionation and high-performance liquid chromatography (HPLC).However, the current mainstream extraction method employs a water-alcohol extraction combined with multi-column chromatography (Meng et al., 2023b),This technique improves the recovery rate and purity of biphenyl components through stepwise extraction and multidimensional chromatographic separation.

#### 4.3.3 Biological activity

##### 4.3.3.1 Anti-skin aging effects

The biaryl compounds and their derivatives found in *Dendrobium officinale* inhibit photoaging of the skin. Biphenyl derivatives are able to regulate superoxide dismutase within the mitochondrial matrix. By lowering its acetylation degree, these compounds effectively boost the antioxidant capacity of SOD. They also convert superoxide anions into less toxic hydrogen peroxide, thereby protecting cells from oxidative stress damage and ultimately alleviating cellular damage caused by the accumulation of reactive oxygen species (ROS) induced by UV-B irradiation (Chen et al., 2022b).

### 4.4 Diphenylstyryl glycosides

Diphenylstyryl glycosides are chemical compounds unique to plants of the Dendrobium genus; more than 30 types have been isolated and identified to date. These ingredients fall into two major categories: monomeric diphenylstyryl glycosides and dimeric ones. The former covers dendrobioside A/B/C, dendrochrysanoside and nobileoside, while the latter mainly includes bisglycosides and cross-linked dimeric derivatives from Dendrobium. Structurally, they possess a basic C6-C2-C6 skeleton and often form glycosides with sugar moieties; some compounds exhibit unique stereochemical configurations.

### 4.5 Other components

In addition, they are rich in bioactive components, including immunomodulatory peptides (dendrobium peptides and cyclic peptide compounds), essential trace elements (zinc, selenium, manganese, etc., with contents highly dependent on growth conditions), and potent antioxidant flavonoids (apigenin and quercetin derivatives).

## 5 Clinical applications

### 5.1 Traditional applications

In traditional Chinese medicine, *Dendrobium officinale* is primarily used to treat conditions such as yin deficiency with internal heat, deficiency of stomach yin, dry mouth and throat, and dry, sore eyes. It is most widely used for treating yin deficiency with internal heat, often in combination with other yin-nourishing herbs such as Ophiopogon japonicus and Scrophularia ningpoensis; For stomach yin deficiency, it is often combined with Saponaria root and Raw Rehmannia root; for dry eyes and improving vision, it is frequently used with Goji berries and Chrysanthemum flowers to enhance the effects of nourishing yin and improving vision (Li et al., 2025c).

### 5.2 Modern clinical research

Current clinical evidence supports the therapeutic potential of *Dendrobium officinale* in chronic metabolic disorders, ocular discomfort, gastric mucosal injury, and immune dysfunction (Zhang et al., 2017; Ling et al., 2022; Chen et al., 2023a; Wang et al., 2025a). Existing clinical observations have verified its effects on blood glucose stabilization, dry eye relief, gastric protection, and immunity regulation, consistent with its traditional yin-nourishing and heat-clearing properties. Nevertheless, formal clinical trials targeting chronic disease treatment and eye health intervention remain limited in scale and standardization. Further standardized clinical validation is therefore required to confirm its dose efficacy, long-term safety, and stable therapeutic outcomes.

Regarding ethical concerns, rigorous ethical review and informed consent procedures are mandatory for all future human clinical investigations. Since most available clinical data are derived from observational adjuvant applications and non-invasive health intervention trials, the ethical risks remain relatively low. However, once formal controlled trials targeting chronic disease treatment are conducted, standardized ethical approval, participant protection, and strict trial registration must be implemented to ensure scientific credibility and compliance with clinical research norms.

## 6 Current status of product development

### 6.1 Health supplements

A variety of health care products made from *Dendrobium officinale* have been developed at present, covering oral solutions, capsules, granules and tablets. Oral preparations stand out among them owing to superior absorption efficiency and convenient administration; capsules offer good stability and portability; granules are easy to prepare and use; and tablets hold a certain market share due to their precise dosing. These products have been developed in a variety of specifications and formulations to meet the needs of different consumer groups.

### 6.2 Functional foods

The development of *Dendrobium officinale* functional foods is becoming increasingly diverse, encompassing various forms such as beverages, candies, herbal teas, and health wines. In the beverage sector, products such as Dendrobium drinks and blended fruit juices have been developed; in the confectionery category, Dendrobium lozenges and gummies have been introduced; herbal tea products mainly include floral teas and blended teas; and in the health wine sector, Dendrobium wines with varying alcohol contents and formulations have been developed.

### 6.3 Cosmetics

Applications in the cosmetics sector primarily include face masks, serums, eye creams, and other skincare products. Facial masks mainly deliver hydration and moisturization, while essences target anti-aging and skin brightening. Eye creams work to ease periocular fatigue and diminish fine lines. Meanwhile, a full range of basic skincare items including day and night creams have also been developed. In formulation design, emphasis is placed on synergistic effects with other functional ingredients to enhance the products’ market competitiveness. In short, **Table 2** provides a clear overview of the various product forms and functional applications of *Dendrobium officinale*, systematically categorizing them into healthcare, cosmetic, and functional food sectors.

**Table 2:** Dendrobium-related biological activities and product development applications.

Application area related to dendrobium	Developed products	Functions/roles played	Main active components on which the products are based	References
Healthcare	Dendrobium Oral Liquid	Boosts immunity	Polysaccharides	(Chen et al., 2021; Fu et al., 2023; Xia et al., 2023)
	Dendrobium Fermented Enzyme Powder	Antioxidant, anti-aging	Polysaccharides, Flavonoids	
	Dendrobium Liver-Protecting Capsules	Liver protection, alcohol detoxification	Alkaloids	
Cosmetics	Anti-aging Facial Mask	Skin repair	Polysaccharides	(Chen et al., 2022b; He et al., 2024; Ren et al., 2025)
	Collagen Essence Serum	Moisturizing, anti-wrinkle	Polysaccharides	
	Whitening Cream	Skin whitening, spot reduction	Polysaccharides, Flavonoids	
	Sunscreen	Anti-aging sunscreen	Bibenzyl compounds	
Functional foods	Dendrobium Probiotic Powder (Solid Beverage)	Regulates gut microbiota	Oligosaccharides, Probiotics	(Liu et al., 2023; Wei et al., 2017; Wu et al., 2023a; Zhou et al., 2024)
	Sleep Aid Gummies	Improves sleep quality	GABA, Alkaloids	
	Sports Drink	Anti-fatigue	Polysaccharides, Minerals	
	Meal Replacement Bar	Satiety, blood sugar control	Dietary Fiber	

This table summarizes the developed products, functional roles, and core active components of Dendrobium in healthcare, cosmetics, and functional foods, based on data from previously published peer-reviewed studies. All information was collated from relevant literature.

## 7 Market status and competition analysis

The industrial chain of *Dendrobium officinale* Kimura et Migo has formed a diversified and multi-track development pattern covering crude raw materials, health food, functional daily food, pharmaceutical deep-processing products, and ornamental horticulture, with distinct developmental maturity, profit margins and market barriers across different segments, forming a multi-level and differentiated industrial ecosystem (Chen et al., 2019). Relevant analysis in this section is based on publicly available official industry information.

At the bottom of the industrial chain, primary processed products including fresh strips, dried strips and common spiral Dendrobium occupy the largest market volume. Nevertheless, this low-end segment suffers from severe product homogenization, insufficient quality control and low industrial added value, accompanied by slow market growth and persistent fierce price competition, resulting in low profit margins for most planting and primary processing enterprises. Such bottlenecks have long been noted as key challenges restricting industrial development (Bao et al., 2024).

In the mid-end market, Dendrobium health care products represented by ordinary Fengdou powder, oral liquid and capsules constitute the main segment of Dendrobium terminal consumption. These health-food-qualified products feature low market access thresholds and extensive offline and online sales channels. However, the sector is plagued by uneven product quality, lack of unified industry standards and scattered market competition, failing to fully release the industrial value of high-quality Dendrobium resources.

Benefiting from the official inclusion of *Dendrobium officinale* in the national medicinal and edible plant catalogue in 2023, emerging functional consumer markets represented by Dendrobium puree, functional beverages and floral tea have achieved the fastest industrial growth rate. Such lightweight and scenario-based products cater to the health needs of young consumer groups, realizing innovative market expansion and becoming a core incremental driver for the sustainable development of the Dendrobium industry. In addition, Dendrobium ornamental potted plants and fresh cut flowers have gradually developed into a niche high-margin track, further enriching the diversified industrial system.

In contrast, the high-end pharmaceutical deep-processing segment represented by *Dendrobium officinale* crystal stands firmly at the top of the industrial value chain. This track requires stringent national drug registration approval, systematic pharmacological and clinical verification, as well as standardized GMP full-process production certification, owning the highest technical, clinical and policy barriers in the whole industry. The pharmacological basis, development characteristics and industrial advantages of pharmaceutical-grade Dendrobium preparations have been systematically elaborated in previous reviews (Tang et al., 2016). The unique pharmaceutical qualification and sufficient clinical evidence endow the products with excellent efficacy stability and market premium capability, which fundamentally differentiates pharmaceutical-grade Dendrobium preparations from ordinary health supplements and primary processed products, forming an absolute oligopolistic market pattern.

Overall, the current *Dendrobium officinale* industry presents a typical unbalanced developmental structure. The primary planting and preliminary processing sectors face serious overcapacity and low profit returns, while the high-value pharmaceutical deep-processing sector is in short supply with ultra-high industry barriers. Meanwhile, emerging functional food and ornamental sectors maintain rapid incremental growth. The coexistence of saturated low-end market, vacant high-end market and booming emerging tracks shapes the unique competitive landscape of the modern Dendrobium industry (Chen et al., 2019).

(Boyan Consulting, 2026; docBoyan Consulting & Market Research Online Network, 2026).

## 8 Trends and outlook

### 8.1 Research directions

Future research will concentrate on the following directions.

#### 8.1.1 Multi-omics and network pharmacology research

Advanced multi-omics strategies and network pharmacology have become indispensable systematic approaches for uncovering the complex molecular mechanisms of medicinal herbs. By integrating transcriptomics, metabolomics, and microbiomics, multi-omics techniques enable comprehensive profiling of key functional genes, differential metabolites, and microbial communities associated with the biosynthesis and bioactivity of Dendrobium active components. Combined with network pharmacology, these tools construct “component–target–pathway” regulatory networks, systematically interpreting the multi-target, multi-pathway, and synergistic pharmacological characteristics of Dendrobium alkaloids, polysaccharides, flavonoids, and bibenzyls (Li et al., 2022; Xu et al., 2023; Yuan et al., 2024). This systematic analytical framework provides powerful technical support for future in-depth mechanism exploration and translational research of *Dendrobium officinale*.

#### 8.1.2 Microbial fermentation technology

Microbial fermentation has emerged as an effective technique to enhance the nutritional and medicinal value of *Dendrobium officinale* products. Functional strains including yeasts and lactic acid bacteria can produce hydrolytic enzymes to degrade macromolecular polysaccharides and proteins into small-molecule compounds with higher bioavailability (Chen et al., 2023a; Yu et al., 2023). This technology also facilitates the biotransformation of alkaloids, bibenzyls and flavonoids, improves nutritional composition and removes anti-nutritional factors, thereby strengthening multiple biological activities. At present, relevant studies mainly focus on polysaccharides and dendrobine, while research on other active components is still insufficient and deserves in-depth exploration.

#### 8.1.3 Molecular targeting and synergistic mechanism exploration

Current pharmacological research on *Dendrobium officinale* is gradually shifting from phenotypic efficacy observation to in-depth mechanistic exploration. Although multi-omics and network pharmacology have preliminarily predicted component–target–pathway relationships, the specific molecular targets of polysaccharides and alkaloids remain insufficiently validated. Moreover, the synergistic interactions among multiple active ingredients are still poorly understood. Future research will focus on identifying accurate functional targets and systematically clarifying the synergistic regulatory mechanisms of different components, which is essential for revealing the multi-pathway pharmacological rules and supporting precise pharmacological interpretation of Dendrobium efficacy.

#### 8.1.4 Genomics, molecular markers and gene editing research

Our bibliometric and keyword mapping analyses show that genomic, gene editing and molecular marker technologies are still rarely applied in current *Dendrobium* studies. The chromosome-level reference genome facilitates the identification of key genes for active component biosynthesis (Niu et al., 2021). Comparative genomics reveals genomic variation and evolutionary adaptation in *Dendrobium* orchids (Chen et al., 2025). Genome-wide identification of regulatory gene families helps illustrate the regulation of secondary metabolism (Li et al., 2025b). Molecular markers have been used for germplasm identification and genetic diversity analysis. The CRISPR/Cas9 system has been established for targeted gene modification in *Dendrobium officinale* (Kui et al., 2017). Future application of these technologies will help reveal the molecular basis of quality formation, accelerate molecular breeding, and provide strong technical support for the standardized production and high-value development of *Dendrobium* products.

#### 8.1.5 Innovative drug delivery and formulation development

The low *in vivo* absorption efficiency and rapid metabolic clearance of natural Dendrobium active components greatly restrict its practical medicinal and functional application. To address this bottleneck, developing novel drug delivery systems and sustained/controlled-release formulations has become a vital technical route for product upgrading. Such innovative formulation strategies can effectively improve the bioavailability of core ingredients, enrich product types, expand application scenarios, and further accelerate the translational transformation of high-value *Dendrobium officinale* products.

### 8.2 Industry development

The industrial expansion of *Dendrobium officinale* is currently constrained by several key bottlenecks limiting large-scale commercialization. First, disparities in geographical origins, cultivation conditions and processing techniques cause unstable active ingredient contents and inconsistent efficacy, undermining market credibility. Second, the absence of unified quality standards, specific evaluation indicators and standardized detection methods impedes cross-regional circulation and systematic quality supervision. Third, insufficient innovative R&D leads to severe product homogenization, single dosage forms and low added value, failing to meet diversified market demands. Fourth, high costs in cultivation, ingredient extraction and production restrict large-scale industrial promotion. Fifth, limited clinical evidence, safety data and mechanistic research weaken the scientific basis and market competitiveness of products. Sixth, inadequate market promotion and vague brand positioning hinder industrial upgrading.

The sustainable industrial development of *Dendrobium officinale* relies on systematic optimization of product layout, quality supervision, industrial chain construction and market regulation, to tackle the aforementioned industrial bottlenecks in a targeted manner. First, standardized cultivation, harvesting and post-processing specifications and full-process traceability platforms should be established to address quality fluctuations caused by variable geographical and cultivation conditions, stabilizing active component contents and ensuring consistent product efficacy. Second, a comprehensive, multi-index, and efficacy-oriented quality evaluation system with unified detection standards for alkaloids, bibenzyls and flavonoid biomarkers needs to be constructed, strengthening the correlation between quality indicators and biological efficacy to support standardized supervision and cross-regional product circulation. Third, differentiated product positioning and customized development for diverse consumer groups can effectively solve the problems of homogeneous structure and low added value, enriching product dosage forms and meeting diversified market demands. Fourth, optimized planting, extraction and production technologies help reduce industrial costs and promote large-scale industrial promotion. Fifth, supplemented systematic clinical verification, safety assessment and in-depth mechanistic research can further consolidate the scientific basis and market competitiveness of Dendrobium products. Sixth, intensified market popularization and clear brand positioning facilitate industrial transformation and upgrading. Furthermore, constructing an integrated “cultivation-processing-R&D-sales” industrial system and improving standardized market regulation and industry self-discipline can further standardize market order and elevate the overall quality threshold of *Dendrobium officinale* products.

## 9 Conclusion

*Dendrobium officinale* and related medicinal Dendrobium species represent economically and pharmaceutically important plants with remarkable health benefits and great industrial potential. Based on bibliometric analysis, we identified several key trends and critical gaps in current research, including unbalanced component studies, insufficient mechanistic interpretation, inconsistent quality control, and a large gap between basic research and industrial translation.

In this review, we systematically sorted out the research progress of Dendrobium from resource characteristics, cultivation conditions, and chemical composition to biological activity and clinical performance, with a focus on the structure–activity–application relationships of polysaccharides, alkaloids, bibenzyls, and other active components. We comprehensively discussed the key links from raw material production, component extraction, and activity verification to product development and industrialization.

On this basis, we put forward forward-looking insights into the future development of Dendrobium research. We emphasized the application value of cutting-edge tools including genomics, gene editing, multi-omics integration, and network pharmacology in revealing quality formation mechanisms, identifying functional genes, and predicting molecular targets. We also highlighted the urgency of establishing multi-index quality evaluation systems, standardized detection methods, and full-process traceability platforms to improve product stability and consistency.

Despite considerable achievements, challenges still exist in germplasm innovation, precise quality control, clinical verification, and high-value product development. This review aims to provide an integrated and updated framework for understanding the research status of Dendrobium and offer valuable guidance for its future mechanistic research, clinical translation, standardized production, and industrial upgrading.

## Data Availability

The original contributions presented in the study are included in the article/supplementary material. Further inquiries can be directed to the corresponding author/s.
